# Physical Activity and Adherence to the Mediterranean Diet among Spanish Employees in a Health-Promotion Program before and during the COVID-19 Pandemic: The Sanitas-Healthy Cities Challenge

**DOI:** 10.3390/ijerph18052735

**Published:** 2021-03-08

**Authors:** Evelia Franco, Jesús Urosa, Rubén Barakat, Ignacio Refoyo

**Affiliations:** 1 Department of Education, Research, and Evaluation Methods, Faculty of Social and Human Sciences, Universidad Pontificia Comillas, 28049 Madrid, Spain; efalvarez@comillas.edu; 2 Sports Department, Faculty of Physical Activity and Sports Sciences—Faculty of Physical Activity and Sports Sciences INEF, Universidad Politécnica de Madrid, 28040 Madrid, Spain; jesus_urosa@hotmail.com (J.U.); ignacio.refoyo@upm.es (I.R.); 3 Social Sciences Applied to Physical Activity, Sport and Leisure Department, Faculty of Physical Activity and Sports Sciences—INEF, Universidad Politécnica de Madrid, 28040 Madrid, Spain

**Keywords:** COVID-19, sedentarism, physical activity, Mediterranean diet, healthy lifestyles

## Abstract

Physical activity and a healthy diet are key factors for avoiding major noncommunicable diseases. The aim of the present study was to analyze how physical activity (PA) and adherence to the Mediterranean diet among employees participating in the Healthy Cities program have been affected during confinement due to the COVID-19 pandemic. The sample was composed of 297 employees from 40 leading companies based in Spain, who participated in the 5th edition of the Sanitas “Healthy Cities” challenge. The participants (148 women and 149 men), aged between 24 and 63 years old (M = 42.76; SD = 7.79) completed the short form of the International Physical Activity Questionnaire (IPAQ) and the PREDIMED (Prevención con Dieta Mediterránea) questionnaire to measure adherence to the Mediterranean diet before and during the pandemic. Pearson χ^2^ tests revealed that workers were more likely to show sedentary behaviors during the pandemic than before (83.5% vs. 66.7%). Additionally, they were more likely to reach high levels of PA (51.2% vs. 64%), and Wilcoxon tests revealed that energy expenditure measured in Metabolic Equivalent of Task (MET) was higher during the pandemic (4199.03 METs) than before (3735.32 METs), due to an increase in moderate PA. Lastly, a higher adherence to a Mediterranean diet during the pandemic (76.4%) than before (54.5%) was reported. The findings of this investigation suggest a positive effect of working from home for employees involved in a health-promotion program, and highlight the relevance of this kind of action among this population.

## 1. Introduction

The health of today’s working population is influenced by their work environment, which has become increasingly sedentary with the rise of the desktop computer. Sedentary jobs currently account for 39% of all jobs in Europe [1]. This is an important public health issue, as evidence is accumulating that sedentary behavior might be associated with undesirable health outcomes [2,3,4]. In this line, there is evidence of a high prevalence of metabolic syndromes among office workers [5], suggesting that sedentary work tasks could be categorized as a hazardous exposure that increases a worker’s risk of adverse health outcomes. In the Spanish context, previous studies have suggested that the prevalence of sitting time among office workers is beyond 6 h a day [6].

Physical inactivity and unhealthy diets are key risk factors for major noncommunicable diseases [7,8]. It explains that in 2004, the World Health Assembly adopted the WHO’s Global Strategy on Diet, Physical Activity and Health [9].

Efforts to promote physical activity (PA) are not surprising, considering that strong evidence demonstrates that compared to less active adult men and women, individuals who are more active exhibit healthier patterns [10,11].

According to the WHO [12], the recommendation for adults to improve health parameters is to perform at least 150 min of moderate-intensity or 75 min of vigorous-intensity aerobic physical activity throughout the week (or an equivalent combination of moderate- and vigorous-intensity activity). Aerobic activity should be performed in bouts of at least 10 min duration and muscle-strengthening activities should be performed involving major muscle groups on two or more days a week. For additional health benefits, the WHO recommends adults should increase their moderate-intensity aerobic physical activity to 300 min per week, or engage in 150 min of vigorous-intensity aerobic physical activity per week, or an equivalent combination of moderate- and vigorous-intensity activity.

In this line, it has been suggested that total PA should be several times higher than these recommendations to achieve larger reductions in the risk of breast cancer, colon cancer, diabetes, ischemic heart disease, and ischemic stroke [13]. In the existing literature, there is evidence that higher volumes of PA are associated with spending less time sitting at work among adult population across different countries [14,15,16] including Spain [6]. This fact might lead us to think that office workers are likely to exhibit lower PA levels than people carrying out other professional tasks. 

On the other hand, diet habits have increasingly become a cause of concern because of their potential consequences for health [17]. In this line, the Mediterranean diet has been linked to a number of health benefits, including reduced mortality and incidence of cardiovascular disease [18] as well as a relatively high level of self-rated health [19]. This diet is abundant in minimally processed plant-based foods, is rich in monounsaturated fat from olive oil, and is lower in saturated fat, meats, and dairy products.

With the literature addressing the assessment of adherence to Mediterranean diet among office workers being scarce, it has been suggested that workers of large companies show moderate adherence to this dietary pattern [20].

### 1.1. The Impact of the COVID-19 Pandemic

In 2020, the world experienced such exceptional circumstances that the usual health determinants were affected at all levels. The spread of the SARS-CoV2 (severe acute respiratory syndrome coronavirus 2), which gave rise to the disease known as COVID-19, led the WHO to declare a global pandemic in March 2020 [21].

Spain has been one of the hardest hit and earliest inflicted countries, with a total of 3,188,553 cases diagnosed, resulting in 69,142 deaths as of February 26 [22]. In an attempt to fight the spread of the virus, the Spanish government approved a period of strict home confinement from 15 March to 2 May 2020. The confinement measures were then gradually relaxed working up until the summer months, but many companies maintained working-from-home (WFH) measures. Some of the immediate consequences of the confinement period were that people had to stay at home more than usual, interrupting their usual activities, and had to change their lifestyle habits [23].

Prolonged quarantine measures have forced businesses to adapt to their workforces being confined at home and no longer able to come into the office. Since the start of mandatory quarantine measures, there has been a significant rise in both public and private sector WFH participation, as well as WFH employee production and working hours [24]. A comparison between Spanish data in 2019 and 2020 revealed that remote work rose from less than 5% to 34% during the quarantine [25].

This shift from office to home working may well have affected healthy habits among workers. Thus, assessing how health risks and benefits of WFH are affected by its sudden, large-scale uptake in the context of COVID-19 is key to best preserving occupational health. Ensuring healthy lives and promoting well-being at all ages, which is essential to sustainable development [26], has become especially relevant in the current global health crisis. This explains the emergence of studies aiming to understand health-related behavioral changes during quarantine [27,28,29,30]. In the Spanish context, only the 66% of the adult population (between 18 and 69 years old) reached the recommendations for PA levels established by the WHO before the pandemic period [31].

### 1.2. Health Promotion among Workers: The Case of Healthy Cities by Sanitas

There is existing evidence of the benefits of health promotion by companies among their employees. For instance, it seems that PA can improve employee-performance at work [32], and can reduce absenteeism [33], and health care costs [34]. This fact explains the interest of companies to introduce corporate health programs [35]. In fact, nearly half of American companies offer some form of support for employees’ wellness [36].

To the best of our knowledge, no previous studies have analyzed how the PA and dietary patterns reported before and during confinement have changed among workers from companies participating in a health promotion program. Given the possibility of new virus outbreaks and thus the continuation of WFH for many people, the spread of WFH to new people seems to be a more than likely future situation. Considering this possibility, it might be of great value to companies to understand the health-related impacts of WFH. This knowledge might be helpful to design their own programs to proactively address the occupational health of their staff in these types of situations in the future. 

The main aim of the present study was to analyze how PA and adherence to the Mediterranean diet among workers participating in the Healthy Cities program have been affected during confinement, i.e., WFH due to the COVID-19 pandemic. As a second goal, we examined whether certain covariates (such as gender, having suffered from COVID-19, living with someone suffering from COVID-19, or having been taking care of others) could have influenced the effect of the confinement on the PA and dietary patterns among participants.

## 2. Methods

### 2.1. Participants

The sample was composed of Spanish office employees participating in the Healthy Cities scheme developed by Sanitas (https://corporativo.sanitas.es/sobre-nosotros/sostenibilidad/healthy-cities/ accessed on 3 March 2021). There were 2491 employees who completed an online survey containing the questionnaires used in the present study before the pandemic. These participants were contacted via e-mail again in May 2020. This time, the questionnaires were answered by 297 subjects, who, therefore, constitute the research sample (148 women and 149 men). The average age of the participants was 42.76 years (standard deviation (SD) = 7.79) ranging from 24 to 63.

### 2.2. Instruments

Information about the participants’ demographic variables, such as gender, age, and the company they belonged to, was gathered in the first data collection. For the second data collection, the participants were also asked whether they had suffered from COVID-19, whether any member of the family they lived with had suffered from the virus, and whether they had been looking after others during confinement. A questionnaire composed of the following validated scales was administered in both moments:

- *Physical Activity*. The Spanish version [37] of the short form of the International Physical Activity Questionnaire (IPAQ; [38]) was used to measure the PA levels of the participants. This questionnaire consists of seven generic items regarding the last seven days, assessing the types and intensity of PA that the participants performed (i.e., vigorous PA, moderate PA, and walking) and the sitting time spent as part of their daily lives. Answers were considered to estimate total PA in metabolic equivalents of task (METs) per week and time spent sitting. The IPAQ defines three categories of PA, namely, “low,” “moderate,” and “high,” according to the WHO’s definitions [9].
- *Adherence to Mediterranean Diet.* The PREDIMED (Prevención con dieta mediterránea) questionnaire was used [39] to measure the adherence to a Mediterranean diet. This instrument consists of 14 items in which participants are asked about their diet habits (e.g., “Do you mainly use olive oil to cook?”). Depending on their answers, participants could score 0 or 1 points for each question. Those who reached nine points in the questionnaire were deemed as Mediterranean diet followers.

### 2.3. Procedure

Sanitas, a leading Spanish health company, developed the Healthy Cities Challenge which was carried out from September 2019 to September 2020 aiming (1) to promote healthy lifestyle habits among the employees belonging to the big Spanish companies who participated in the programme and (2) to generate a financial donation by Sanitas to an urban regeneration project, in a Spanish city, as a vehicle to develop more areas where people can go to carry out PA [40].

As for the first aim, workers belonging to the participant companies were invited to participate in a PA challenge, with the option to then access health-related offline events and online workshops. The challenge was to walk 10,000 steps a day, with their steps recorded through a digital platform, specifically designed for this program. Participants could follow the progress of their individual challenge as well as the overall challenge, and access free professional health and wellbeing counselling.

A quantitative research approach with a pre–post design was adopted in this study. An online platform was used to create and distribute the questionnaire. The initial questionnaire was administered in October 2019 and the second questionnaire was administered in May 2020. In both cases, the questionnaire was disseminated through platforms available for the staff of companies participating in the Healthy Cities Project.

To assess whether the participants reached the PA levels, recommendations by WHO [9] and by Kyu et al. [13] were considered. According to the WHO [9], the recommendation for adults to improve health parameters is to perform at least 150 min of moderate-intensity or 75 min of vigorous-intensity aerobic physical activity throughout the week (or an equivalent combination of moderate- and vigorous-intensity activity reaching 600 MET of physical activity. Aerobic activity should be performed in bouts of at least 10 min duration and muscle-strengthening activities should be performed involving major muscle groups on two or more days a week. For additional health benefits, the WHO recommends adults should increase their moderate-intensity aerobic physical activity to 300 min per week, or engage in 150 min of vigorous-intensity aerobic physical activity per week, or an equivalent combination of moderate- and vigorous-intensity activity. According to Kyu et al. [13], an increase from 600 to 3600 MET would significantly reduce the risk of noncommunicable diseases.

The time required to answer the questionnaire was approximately 20 min. The participants were able to answer the questionnaire at different times. The responses to the questionnaires were stored in an online database to which the authors had access. All of the participants were treated in agreement with the ethical guidelines of the American Psychological Association [34].

### 2.4. Data Analysis

First, a Kolmogorov–Smirnov test was performed to verify the normality of the data, which showed that it was non-normally distributed (*p* < 0.05). Thus, Wilcoxon tests were performed to analyze possible differences in PA between before and during the pandemic. Then, a series of χ2 tests were performed to test for any potential effects of the pandemic on PA and Mediterranean diet-related variables. Finally, additional χ2 tests were performed to investigate whether the effects of the pandemic could have differed according to gender, having suffered or not from COVID-19, having cohabited with someone suffering from COVID-19, or having been looking after others.

## 3. Results

Table 1 shows the participant distribution according to the covariables included in the study.

As shown in Table 2, the Wilcoxon tests revealed that participants significantly increased their level of total PA during the pandemic (3735.32 METs vs. 4199.03 METs). While no significant differences were found in terms of vigorous activity or walking, the METs spent on moderate activity was remarkably higher during the pandemic than before (643.70 vs. 971.53, respectively).

Table 3 shows the distribution of participants in terms of PA levels according to the WHO, and whether they reached PA levels recommended by Kyu et al. [10], showed sedentary behavior, and stuck to a Mediterranean diet. A Pearson χ2 test, together with an analysis of the adjusted residuals, revealed that the ratio of participants reporting medium levels of PA decreased during the pandemic, while the ratio of participants showing high levels of PA increased during the pandemic. As for reaching the recommended PA levels, although the total number of participants was equally distributed both before and during the pandemic, the analysis of the adjusted residuals showed that many participants (*n* = 50) changed from not reaching recommended levels to reaching them or vice-versa. In terms of sedentarism, the ratio of sedentary participants significantly increased during the pandemic. Finally, more participants reported adherence to a Mediterranean diet during the pandemic.

Table 4 presents the χ2 test results for the distribution of participants in PA and Mediterranean diet-related variables according to gender. It can be noted that the effect of the pandemic on the distribution of participants is similar for men and women in terms of PA levels, sedentarism, and Mediterranean diet adherence. However, regarding reaching the recommended PA levels, while the number of men reaching these recommended levels decreased, there were more women reaching the recommended levels during the pandemic than before.

Table 5 presents the χ2 test results for the distribution of participants who did and did not suffer from COVID-19 in terms of PA and Mediterranean diet-related variables. It can be seen that suffering from COVID-19 had a negative impact on PA levels (the number of participants reporting high levels of PA in this group decreased). The ratio of participants reaching the recommended levels also decreased during the pandemic among those suffering from COVID-19, while it increased among the remaining participants. In terms of sedentary behavior, all of the participants suffering from COVID-19 reported being sedentary during the pandemic.

Table 6 presents the χ2 test results for the distribution of participants who did or did not cohabit with someone suffering from COVID-19 in terms of PA and Mediterranean diet-related variables. It can be noted that the effect of the pandemic on the distribution of participants is similar for both groups in all variables.

Table 7 presents the χ2 test results for the distribution of participants in terms of PA and diet-related variables. Results are presented separately for people who were looking after others and people who were not looking after others during the pandemic. As shown, the effect of the pandemic on the distribution of participants was similar for both groups in terms of the levels of PA and sedentarism. However, as for reaching the recommended PA levels, while the number of participants reaching these recommended levels decreased in the group who were not looking after others during the pandemic, it increased in the group of people who were looking after others. Finally, it is worth mentioning that the rise in the number of participants sticking to a Mediterranean diet was more pronounced in the group of participants looking after others.

## 4. Discussion

The main aim of the present study was to analyze how PA and adherence to a Mediterranean diet among workers participating in the “Healthy Cities by Sanitas” program have been affected during confinement when WFH due to the COVID-19 pandemic. 

Overall, in terms of PA, the findings of the present study suggest that Spanish employees participating in a health promotion program have shown higher levels of both sedentarism and PA during the pandemic than before. As for the dietary patterns, a higher adherence to a Mediterranean diet during confinement when compared to previous habits was found.

### 4.1. Sedentary Lifestyle

During the pandemic, the Spanish government implemented severe quarantine measures. While these measures are highly commendable and critical to mitigate the spread of COVID-19, they may have resulted, according to existing findings, in inducing unhealthy behaviors, such as a sedentary lifestyle [27,41]. Our results are in line with this previous evidence and raise some concern about the prospects of the Spanish population’s health considering the existence of an association between sedentary behavior and certain maladaptive health outcomes, such as morbid obesity, a higher number of chronic conditions, higher disability levels, or a worse health status in terms of mobility [42]. Although there is no evidence of the lasting impact that this global crisis may have on sedentary behaviors, previous research has suggested that a big crisis due to natural disasters can negatively impact PA patterns for three years after the disaster [43]. Considering the above, it would be interesting to implement PA promotion programs not only during the pandemic, but also during the subsequent period.

As for how the virus might have affected sedentarism, it is worth noting that 100% of the participants who reported having suffered from COVID-19 reported being sedentary during the pandemic. It is worth mentioning that the severity of the disease (whether participants were in hospital or not, or whether they suffered from further complications) was not reported in this study and thus we should be cautions in the interpretation of these findings. However, the higher levels of sedentary behavior among participants suffering from COVID-19 is not surprising given that this virus primarily causes respiratory symptoms [44], which might have prevented patients from getting involved in any kind of PA [45]. COVID-19 also seems to be related to cardiovascular conditions [46,47]. In light of the fact that moderate PA might improve outcomes following respiratory viral infection and cardiovascular diseases [48,49], future research should examine how PA might affect COVID-19 patients’ outcomes and should explore what type of practice would be more recommendable for this population. 

### 4.2. Physical Activity

As has been previously pointed out, PA and sedentary behaviors are not the opposite of one another [50]. Individuals are considered to be active when they reach PA recommendations for their age, which does not prevent them from also devoting a significant part of their time to sedentary behaviors. In other words, individuals can be classified as both active and sedentary. Office workers are a good demonstrative example of sedentariness, as they spend a considerable part of their time seated in front of a computer screen. This defines them as highly sedentary, while they may or may not reach their aged-related PA recommendations outside of work [51].

Surprisingly, our results also suggest that Spanish employees taking part in a health promotion programme might have been more active during the pandemic than before. While there have been several studies addressing PA recommendations in times of pandemics, the literature is scarce regarding studies analyzing how pandemic and quarantine measures affect PA levels.

Our results differ to the findings by López-Bueno et al. [28], which revealed that Spanish adults were more likely to reach PA recommendations before than during lockdown measures due to COVID-19. This difference might be explained if participants in the aforementioned study, recruited through general social media, came from different professional backgrounds to those in Sanitas’ Health Cities challenge. 

The fact that our study focused on employees who had voluntarily signed up to a health challenge, in companies that proactively promoted healthy lifestyle, and received continued health assessment during the pandemic as part of the challenge, might explain why their PA did not decrease. 

On the other hand, while the data in the study by Álvarez-Bueno et al. [52] were collected between 22 and 29 March, starting on the seventh official day of the government-enacted national confinement, our second wave of data were collected during the month of May. While restrictions were very strict in March, they were alleviated from early May onward [53] which meant that many people resumed outdoor PA with a renewed enthusiasm, given its prohibition for the previous 7 weeks.

The findings of the present study also suggest that total PA seems to have increased due to a higher METS expenditure in moderate PA. However, METS expenditure in both vigorous PA and walking does not seem to have been affected by the pandemic. In this vein, the studies carried out by Tison et al. [54], Galle et al. [55], and Bourdas and Zacharakis [56] suggest an important decrease in daily mean steps within the days following the pandemic declaration.

As mentioned before, it is our belief that the partial lifting of the restrictions in May, when our data were gathered, could have explained this difference between previous evidence and our results. From this, we hypothesize that the stricter the confinement measures, the more likely it will be that PA patterns are affected by the situation. Furthermore, it must be pointed out that certain adults might have even increased their moderate activity levels. In this regard, there have been efforts to help individuals be physically active during COVID-19 that should be applauded. The Healthy Cities platform provided by Sanitas adapted its health coaching and recommended activities during confinement so that participants could adapt their step count through different types of activities. Sanitas also provided mental health support during this period to keep motivation high. The American College of Sports Medicine released information on how to remain active during COVID-19 [57]. Numerous fitness centers have also been posting free online workout routines to help people remain active at home [58,59,60,61]. While the offer of these kinds of activities is varied in nature (e.g., yoga, dance, calisthenic workouts), they are likely to display moderate intensity routines. These initiatives might have contributed to the higher levels of moderate activity shown by the participants of this study.

### 4.3. Mediterranean Diet

As has been previously pointed out, it could have been expected that during the pandemic, our diet would have taken a step back from being a healthy diet rich in fresh food to one containing foods with a long shelf life [62]. The reasons supporting this belief are both the threat of a potential food shortage and that a typical response to chronic stressful situations is the consumption of energy-dense foods [63]. However, our results show that the adherence to a Mediterranean diet was higher during the pandemic than before. This fact is not surprising in light of recent studies addressing both dietary and cooking habits. In this vein, Rodríguez-Pérez et al. [64] suggested that COVID-19 confinement in Spain has led to the adoption of healthier dietary habits reflected in a higher adherence to a Mediterranean diet. As was pointed out, devoting more time to both cooking and eating when WFH compared to when working at the office might explain why people have been more likely to engage in healthier diet behaviors [65].

Our results also suggest that people who have been looking after others during confinement were more likely to switch their dietary habits toward a Mediterranean diet. Participants looking after others are likely to be in charge of either children or elderly relatives and, thus, living with them. Living in the family home, compared to living alone, has been associated with a higher adherence to a Mediterranean diet both during the pandemic [64] and in ordinary times [66]. In light of this previous evidence, it could be suggested that family life might foster healthier dietary patterns and that efforts should be made to promote healthier habits among people living alone.

The main strength of the present study is having data gathering just before the pandemic, when it was not possible to foresee that situation. This allowed us to compare the study variables before and after the pandemic. Furthermore, it is focused on a specific population (Spanish employees attending a health promotion program) which contributes to the sample homogeneity. As stated in the next paragraph, this could be both a strength and a limitation. 

This study has some limitations that must be mentioned. While this is one of the very few studies assessing the impact of the pandemic with data gathered both before and during the pandemic, only 297 participants out of the initial 2491 completed the second wave of questionnaires. Second, our sample represents a specific population group, i.e., employees participating in a health promotion program, and the findings cannot be extended to the whole population. The health promotion program that the participants were taking part of could have partially explained the changes observed. It would be interesting for future studies to explore the effects of such programs when under different sanitary conditions. On the other hand, the present study does not provide an in-depth description about people suffering COVID-19 (i.e., to what extent did the symptoms prevent the participants from taking part in PA?). It would be interesting for further studies to address the impact of COVID-19 on PA levels according to the severity of the disease. Lastly, the present study relies on self-reported measures to assess PA. Findings in this respect should be interpreted cautiously, since there is previous evidence suggesting that the IPAQ might underestimate sitting and overestimate time spent in almost all PA intensities [67]. In this respect, IPAQ may not be precise enough given that people who are not familiar with the perception of PA intensities could wrongly interpret the sort of activity they take part in. Therefore, it would be interesting for future studies to address the evaluation of PA levels through objective measures. 

## 5. Conclusions and Practical Implications

The findings of this investigation highlight the need for PA and diet promotion interventions among employees. Sedentary behaviors in this population seem to have been exacerbated during the pandemic. Considering the negative consequences associated with sedentary lifestyles, it could be beneficial for companies to work on the design of strategies aiming to reduce the time spent sitting down by their employees. As for PA promotion, it is suggested that the implementation of initiatives offering employees the possibility of exercising at home could lead to a greater energy expenditure in moderate activities. These measures could be especially expedient if a second wave of the pandemic forces office workers to work from home again or if companies decide to implement WFH for other reasons. With regard to dietary habits, it seems that when working at the office, people are less likely to engage in healthy dietary patterns than when WFH. The implementation of WFH or of certain initiatives, such the organization of work in continuous shifts or the implementation of healthy food offers in the workplace, could help improve the adherence to a healthier diet, such as the Mediterranean one, among workers.

## Figures and Tables

**Table 1:** Distribution of men and women according to their contact with COVID-19 and whether they took care of others.

	Men *N* = 149	Women *N* = 148
*Suffering from COVID-19*		
No	138 (92.6%)	142 (95.9%)
Yes	11 (7.4%)	6 (4.1%)
*COVID-19 contact*		
No	124 (83.2%)	112 (75.2%)
Yes	25 (16.8%)	36 (24.8%)
*Looking after others*		
No	67 (45%)	83 (56.1%)
Yes	82 (55%)	65 (43.9%)

**Table 2:** Effect of confinement on metabolic equivalent of tasks (METs) spent through different types of physical activity.

	Before Pandemic M (DT)	During Pandemic M (DT)	Z	*p*	Cohen’s
Vigorous activity	2001.61 (1933.23)	2045.93 (1770.78)	−1.18	0.238	0.01
Moderate activity	643.70 (910.35)	971.53 (1206.04)	−4.63	0.001	0.19
Walking	1181.58 (1227.62)	1090.00 (1008.29)	−0.271	0.786	0.01
Total activity	3735.32 (2811.95)	4199.03 (2975.91)	−2.74	0.006	0.15

**Table 3:** Distribution of the participants among the different categories of the study variables before and during the pandemic.

	Before Pandemic	During Pandemic	(d.f.) Pearson χ2
*Physical activity levels (WHO)*			
Low	19 (6.4%)	17 (5.7%)	(4) 32.35 ***
Medium	126 (42.4%)	90 (30.3%)	
High	152 (51.2%)	190 (64%)	
*Recommended PA levels*			
Not reached	148 (49.8%)	148 (49.8%)	(1) 31.68 ***
Reached	149 (50.2%)	149 (50.2%)	
*Sedentarism*			
No	99 (33.3%)	49 (16.5%)	(1) 46.97 ***
Yes	198 (66.7%)	248 (83.5%)	
*Mediterranean diet adherence*			
No	135 (45.5%)	70 (23.6%)	(1) 22.26 ***
Yes	162 (54.5%)	227 (76.4%)	

Note. *** *p* < 0.001.

**Table 4:** Effect of confinement on the distribution of participants in terms of PA and adherence to a Mediterranean diet according to gender.

	Men			Women		
	Before	After	(d.f.) Pearson χ2	Before	After	(d.f.) Pearson χ2
*Physical activity levels (WHO)*						
Low	8 (5.7%)	10 (6.7%)	(4) 22.99 ***	11 (7.4%)	7 (4.7%)	(4) 12.33 *
Medium	52 (34.9%)	38 (25.5%)		74 (50.0%)	52 (35.1%)	
High	89 (59.7%)	101 (67.8%)		63 (42.6%)	89 (60.1%)	
*Recommended PA levels*						
Not reached	60 (40.3%)	69 (46.3%)	(1) 14.11 ***	88 (59.5%)	79 (53.4%)	(1) 16.29 ***
Reached	89 (59.7%)	80 (53.7%)		60 (40.5%)	69 (46.6%)	
*Sedentarism*						
No	57 (38.3%)	30 (20.1%)	(1) 27.71 ***	42 (28.4%)	19 (12.8%)	(1) 17.20 ***
Yes	92 (61.7%)	119 (79.9%)		106 (71.6%)	129 (87.2%)	
*Mediterranean diet adherence*						
No	75 (50.3%)	36 (24.2%)	(1) 5.06 *	60 (40.5%)	34 (23%)	(1) 19.93 ***
Yes	74 (47.7%)	113 (75.8%)		88 (59.5%)	114 (77%)	

Note. * *p* < 0.05, *** p < 0.001.

**Table 5:** Effect of confinement on the distribution of participants in terms of PA and adherence to a Mediterranean diet depending on having suffered or not from COVID-19.

	Not Suffering from COVID-19			Suffering from COVID-19		
	Before	After	(d.f.) Pearson χ2	Before	After	(d.f.) Pearson χ2
*Physical activity levels (WHO)*						
Low	19 (6.8%)	16 (5.7%)	(4) 29.89 ***	0 (0%)	1 (5.9%)	(2) 8.74 *
Medium	123 (43.9%)	85 (30.4%)		3 (17.6%)	5 (29.4%)	
High	138 (49.3%)	179 (63.9%)		14 (82.4%)	11 (64.7%)	
*Recommended PA levels*						
Not reached	146 (52.1%)	134 (47.9%)	(1) 31.66 ***	2 (11.8%)	9 (52.9%)	(1)2.02
Reached	139 (49.6%)	141 (50.4%)		15 (88.2%)	8 (47.1%)	
*Sedentarism*						
No	94 (33.6%)	49 (17.5%)	(1) 46.84 ***	5 (29.4%)	0 (0%)	
Yes	186 (66.4%)	231 (82.5%)		12 (70.6%)	17 (100%)	
*Mediterranean diet adherence*						
No	127 (45.4%)	67 (23.9%)	(1) 19.29 ***	8 (47.1%)	3 (17.6%)	(1) 4.10 *
Yes	153 (54.6%)	213 (76.1%)		9 (52.9%)	14 (82.4%)	

Note. * *p* < 0.05, *** *p* < 0.001.

**Table 6:** Effect of confinement on the distribution of participants in terms of PA and adherence to Mediterranean diet depending on having cohabited or not with people suffering from COVID-19.

	No COVID-19 Contact			COVID-19 Contact		
	Before	After	(d.f.) Pearson χ2	Before	After	(d.f.) Pearson χ2
*Physical activity levels (WHO)*						
Low	16 (6.8%)	14 (5.9%)	(4) 33.27 ***	3 (4.9%)	3 (4.9%)	(4) 7.3
Medium	104 (44.1%)	74 (31.4%)		22 (36.1%)	16 (26.2%)	
High	116 (49.2%)	148 (62.7%)		36 (59%)	42 (68.9%)	
*Recommended PA levels*						
Not reached	122 (51.7%)	118 (50%)	(1) 21.99 ***	26 (42.6%)	30 (49.2%)	(1) 10.35 ***
Reached	114 (48.3%)	118 (50%)		35 (57.4%)	31 (50.8%)	
*Sedentarism*						
No	82 (34.7%)	45 (19.1%)	(1) 40.84 ***	17 (27.9%)	4 (6.6%)	(1) 4.73 *
Yes	154 (65.3%)	191 (80.9%)		44 (72.1%)	57 (93.4%)	
*Mediterranean diet adherence*						
No	111 (47%)	58 (24.6%)	(1) 14.85 ***	24 (39.3%)	12 (19.7%)	(1) 7.96 **
Yes	125 (53%)	178 (75.4%)		37 (60.7%)	49 (80.3%)	

Note. * *p* < 0.05, ** *p* <0.01, *** *p* < 0.001.

**Table 7:** Effect of confinement on the distribution of participants in terms of PA and adherence to a Mediterranean diet depending on having looked after others or not during the pandemic.

	Not Looking after Others			Looking after Others		
	Before	After	(d.f.) Pearson χ2	Before	After	(d.f.) Pearson χ2
*Physical activity levels (WHO)*						
Low	8 (5.3%)	10 (6.7%)	(4) 26.95 ***	11 (7.5%)	7 (4.7%)	(4) 11.57 *
Medium	62 (41.3%)	43 (28.7%)		64 (43.5%)	47 (32%)	
High	80 (53.3%)	97 (64.7%)		72 (49%)	93 (63.3%)	
*Recommended PA levels*						
Not reached	67 (44.7%)	77 (51.3%)	(1) 26.30 ***	81 (55.1%)	71 (48.3%)	(1) 8.68 **
Reached	83 (55.3%)	73 (48.7%)		66 (44.9%)	76 (51.7%)	
*Sedentarism*						
No	43 (28.7%)	21 (14%)	(1) 26.97 ***	56 (38.1%)	28 (19%)	(1) 19.98 ***
Yes	107 (71.3%)	129 (86%)		91 (61.9%)	119 (81%)	
*Mediterranean diet adherence*						
No	74 (49.3%)	49 (32.7%)	(1) 16.96 ***	61 (41.5%)	21 (14.3%)	(1) 4.20 *
Yes	76 (50.7%)	101 (67.3%)		86 (58.5%)	126 (85.7%)	

Note. * *p* < 0.05, ** *p* <0.01, *** *p* < 0.001.

## Data Availability

Not applicable.
